# Prevalences of mental distress and its associated factors in unaccompanied refugee minors in Germany

**DOI:** 10.1007/s00787-021-01926-z

**Published:** 2021-12-17

**Authors:** E. Höhne, T. Banaschewski., M. Bajbouj, K. Böge, T. Sukale, I. Kamp-Becker

**Affiliations:** 1 Department of Child and Adolescent Psychiatry, Psychosomatics and Psychotherapy, Faculty of Human Medicine, University Hospital of Marburg and Philipps-University Marburg, Marburg, Germany; 2 Department of Child and Adolescent Psychiatry and Psychotherapy, Central Institute of Mental Health, Medical Faculty Mannheim/Heidelberg University, Mannheim, Germany; 3 Department of Psychiatry and Psychotherapy, Campus Benjamin Franklin, Charité-Universitätsmedizin Berlin, corporate member of Freie Universität Berlin, Humboldt-Universität Zu Berlin, and Berlin Institute of Health, Berlin, Germany; 4 Department of Child and Adolescent Psychiatry and Psychotherapy, University of Ulm, Ulm, Germany

**Keywords:** Unaccompanied refugee minors, Depression, Mental health, Risk and resilience, Prevalence

## Abstract

Prevalences for mental disorders within minor refugees are comparatively high and heterogeneous. To reduce heterogeneity and identify high-risk subgroups, we compared unaccompanied refugee minors (URM) to accompanied refugee minors (ARM) regarding depressive symptoms and mental distress. Furthermore, we examined associative factors of mental distress in URM on a broad scale. We conducted a survey with a cross-sectional design in four German University hospitals. The sample consisted of *n *= 172 URM and *n *= 52 ARM aged 14–21. Depressive symptoms were assessed via the Patient Health Questionnaire (PHQ-9). Mental distress was assessed by the Refugee Health Screener (RHS-15). Mann–Whitney test was used to examine differences between URM and ARM. Associated factors of mental distress were evaluated via a stepwise multiple regression analysis. URM showed significantly higher mean scores for PHQ-9 (*p *< .001) and RHS-15 (*p *< .001) compared to ARM indicating medium effect sizes. Furthermore, URM were significantly more likely to surpass the cut-off for depression (61.6% vs. 30.8%) and overall mental distress (81.4% vs. 53.8%) compared to ARM. The factors *Number of stressful life events (SLE), Female gender*, and *Fear of deportation* were found to be associated with an increased mental distress in URM, whereas *Weekly contact to a family member, School attendance,* and *German language skills* were accompanied with lower distress scores. All six factors accounted for 32% of the variance of mental distress in URM (*p *< .001). Within minor refugees, URM are a highly vulnerable subgroup, which should receive particular attention and more targeted measures by health authorities. Our results indicate that these measures should comprise a rapid promotion of family contact, school attendance, language acquisition, and the fast processing of asylum applications. However, the cross-sectional design limits the interpretability of the results.

**Supplementary Information:**

The online version contains supplementary material available at 10.1007/s00787-021-01926-z.

## Introduction

In 2019, more than 30 million people were considered as refugees or asylum-seekers worldwide [1]. With 1.1 million, Germany is one of the biggest host countries for refugees. About 40% of all displaced people are minors [1]. Most minor refugees have to face multiple burdens before, during, and after their flight, while still facing common adolescent developmental tasks. Due to these various stressors, minor refugees show higher prevalences of psychological distress and emotional problems than non-displaced children [2, 3]. Prevalence rates for depression, as the most common psychiatric disorder in minor refugees, range from 10.3 to 32.8% [4] compared to 2–9% in non-refugee adolescents [5]. Yet, prevalence estimates among minor refugees range considerably in these studies. This striking heterogeneity might be explained by different subgroup distributions in the investigated refugee samples [6]. International reviews suggest that especially unaccompanied refugee minors (URM) represent a highly vulnerable refugee subgroup [7, 8]. However, only a few studies have evaluated distress measures separately for URM and accompanied refugee minors (ARM) with ambiguous results [9, 10]. Due to the inconclusive results of prior research and its important role for preventive measures within a burdened health care system, we aimed to contribute to this field of research by comparing URM and ARM regarding their general mental distress level and, in particular, their level of depressive symptoms. According to emerging evidence, we assumed URM to show higher levels of mental distress and depressive symptoms [11].

Even within the presumed subgroup of URM, prevalence rates can differ enormously. For example, a review reported prevalences for psychiatric disorders in URM to range between 20 and 82% [12]. Moreover, considering the load of burdens most URM experience, many of them show a remarkable resilience [13]. To gain more insight in the driving forces of symptom development, the identification of associated factors for mental distress within URM has received increasing attention lately [14]. To obtain an overview, our research group recently conducted a systematic review on this issue [15]. Since the included studies only investigated a fragmentary set of associated factors, a sequencing of factors according to their mental health impact was difficult. Furthermore, most studies focused their research on pre-flight risk factors (e.g., flight duration, gender, and country of origin) rather than investigating modifiable post-flight factors such as family contact, language acquisition, or school attendance. Additionally, all of the included German studies showed considerably small sample sizes [10, 16–18]. To meet the demand for current and comprehensive information about determinants of mental distress in URM, we aimed to investigate associated factors and sequence them according their impact on the basis of the largest sample of URM in Germany. To achieve this, we created a screening tool (MEHIRA-SQ) comprising all relevant associated factors identified in our previous systematic review [15]. By including a large number of post-flight associated factors, we aimed to provide relevant information for health authorities regarding preventive measures.

## Methods

### Procedure

This study used the screening data of four outpatient clinics for child and adolescent psychiatry which took part in the MEHIRA study, a multicentric RCT for refugees with affective disorders in Germany [19]. Data collection lasted from April 2018 to September 2019. The study was approved by the Ethical Committee of the Charité-Universitätsmedizin Berlin and by the local ethics committees of all participating medical faculties. The MEHIRA study was registered in Clinical Trails.gov (registration number: NCT03109028). Recruitment took place in local schools, residential group accommodations for refugees, and outpatient clinics of the participating university hospitals. For inclusion in our investigation, participants had to be refugees or asylum-seekers as defined by the United Nations High Commissioner for Refugees [20], aged between 14 and 21 years with sufficient language skills and literacy in Arabic, Farsi/Dari, German, or English. Exclusion criteria were current psychotic symptoms, a degenerative disorder, and/or an acute risk of suicidality. Prior to the screening process, participants were informed about the objectives, content, and risks of the MEHIRA study and the anonymous processing of the data. A written consent (as required in Arabic, Farsi/Dari, German, or English) was obtained by all participants and, if under the age of 18 years, by a legal guardian. All measures were carried out in face-to-face settings (e.g., in outpatient clinics) or in group administration (e.g., in schools and refugee accommodations). In case of difficulties in understanding or need for psychological support, trained psychologists assisted during group administration. All questionnaires were available in Arabic, Farsi, English, and German.

### Measures

#### Refugee health screener-15 (RHS-15)

The level of mental distress was assessed via the Refugee Health Screener 15 (RHS-15). The RHS-15 is a common 15-item screening instrument for newly arrived refugees aged ≥ 14 years to assess symptoms of anxiety, depression, and trauma-related disorders over the last month on a five-point Likert scale in n [21]. Cut-off for a significant level of mental distress is a total sum score (item 1–14) of ≥ 12. The reliability of the RHS-15 is good with an internal consistency Cronbach coefficient alpha of 0.93 [21].

#### Patient health questionnaire (PHQ-9/PHQ-A)

Symptoms of depression were assessed via the PHQ-9. It is a brief and widely used 9-item self-report instrument based on the DSM-IV to assess frequency of depressive symptoms within the last 2 weeks on a four-point Likert scale [22]. The total score can range from 0 to 27 with a clinical cut-off ≥ 10. Internal consistency of the PHQ-9 can be considered high, with a Cronbach’s alpha of 0.86–0.89 [22]. Adolescents under the age of 18 years filled out the PHQ-A, respectively, a slightly modified, highly comparable and well-validated version of the PHQ-9 for adolescents [23].

#### MEHIRA screening questionnaire (MEHIRA-SQ)

Socio-demographic information and potential associated factors of mental distress were assessed with the MEHIRA Screening Questionnaire (MEHIRA-SQ). This 15-item questionnaire was developed for the purpose of this study by two authors (EH, IKB) through multiple feedback loops on the basis of findings of our previous systematic review [15]. It briefly and concisely assesses status of accompaniment, reasons for migration, and 14 empirically supported potential associated factors of mental distress in URM, namely *Weekly contact with a family member (*via media devices*)*, *Number of SLE, Gender, Fear of deportation, School attendance, German language skills, Iimportance of religion, Duration of flight, Age, Country of origin, Time spent in host country, Having friends in the host country*, *Having a trusted person*, and *Sharing a room*. The MEHIRA-SQ is built up like an anamnesis check-list in clinical practice. For higher usability, items are assessed via different methods depending on their content (such as dichotomously, several possible answers, open answer, and Likert scale). Translation was performed through language processors of the MEHIRA committee. The English version of the MEHIRA-SQ is attached in the Supplemental A.

### Statistical analyses

For differentiation between URM and ARM, we assessed the accompaniment by parents throughout the flight via the MEHIRA-SQ. Differences in sample characteristics between URM and ARM were investigated using ANOVA for continuous outcomes, and Chi-square tests for categorical variables. To examine the relation between status of accompaniment (URM vs. ARM) and prevalence rates for mental health outcomes, a Chi-square test was performed. Differences between URM (*n *= 172) and ARM (*n *= 52) regarding their RHS-score and PHQ-score were examined with the Mann–Whitney *U *test due to non-normally distribution of the outcome measures (Shapiro–Wilk test: *W* = 0.96; *p *< 0.001). A two-sided asymptotic alpha level of 0.05 was used for all tests due to diverging sample sizes. Standardized effect sizes (Cohen’s *d*) were calculated for both group comparisons [24]. For predictor analyses (*n *= 172 URM), 14 items of the MEHIRA-SQ were included in a stepwise multiple regression analysis (inclusion criteria *p *< 0.05) with RHS-score as an outcome measure for mental distress. Earlier power analyses yielded in a necessary sample size of *n *= 162 URM for a statistical power of 0.8 (1 − β), given the formula *n *≥ 50 + 8 m [25]. Pre-analysis confirmed the absence of multicollinearity (variance inflation factor < 2) and heteroscedasticity (visual inspection of scatter plot) for all investigated associated factors. Analysis was performed using IBM SPSS statistics version 22.

## Results

### Socio-demographic characteristics

A total of *n *= 231 minor refugees participated in the study. Seven minor refugees did not provide information about their accompaniment status and had to be excluded from further evaluation. Thus, the sample comprised *n *= 172 URM and *n *= 52 ARM with an average age of 18.6 years (SD = 1.59). The majority of the participants were male (85.27%), and migrated from Afghanistan (35%) or Syria (34%) due to war in their country of origin (63%), which is representative for URM in Germany [26]. URM differed significantly from ARM regarding *Gender, Time spent in host country, Country of origin, Number of SLE, School attendance*, and *Reasons for migration*. Detailed sample characteristics for each subgroup are presented in Table 1.

**Table 1:** Sample characteristics for URM and ARM

	URM (*n *= 172)	ARM (*n *= 52)	*p* value
Age in years, M (SD)	18.16 (1.51)	18.13 (1.76)	.93
Gender (%)			< .01
Male	89.5	71.2	
Female	10.5	28.8	
Time spent in host country in month M (SD)	30.41 (12.64)	24.56 (17.72)	< .01
Country of origin (%)			< .001
Afghanistan	42.4	13.5	
Syria	25.6	65.4	
Somalia	7.6	0.0	
Eritrea	8.1	0.0	
Other (< 5%)	16.3	21.1	
Number of SLE, *M* (SD)	3.72 (1.89)	2.04 (1.44)	< .001
Duration of flight in weeks, *M* (SD)	38.89 (66.95)	39.64 (80.82)	.95
Having friends in host country (%)	85.5	88.5	.49
School attendance in host country (%)	84.3	96.2	< .05
Reason for migration (%)			
War	58.7	80.8	< .01
Natural disaster	1.2	0.0	.99
Economic crisis	8.1	15.4	.18
Individual situation	25.0	21.2	.71
Political/religious persecution	43.6	26.9	< .05
Social situation	12.8	23.1	.08
Other	17.4	7.7	.12
PHQ-Score, Mdn (SD)	25.56 (14.05)	15.17 (12.76)	< .001
RHS-Score, Mdn (SD)	12.04 (6.32)	8.00 (6.97)	< .001

### Prevalences and symptom severity

URM (81.4%) were more likely to surpass the clinical cut-off for depression than ARM (53.8%). A Chi-square test indicated that this relation between status of accompaniment (URM vs. ARM) and prevalence for depression was statistically significant [*χ*^2^ (1, *n *= 224) = 16.16, *p *< 0.001]. Regarding symptom severity of depression, PHQ-9 scores of URM (Mdn = 25.56; SD = 14.05) were higher than those of ARM (Mdn = 15.17; SD = 12.76). A Mann–Whitney test indicated that this difference was statistically significant (*U* = 2837.50, *Z* =  − 3.996 *p *< 0.001) with an upper medium effect size *d* = 0.703 (95% CI 0.437 to 1,073) according to Cohen [27].

URM (61.6%) were more likely to surpass the clinical cut-off for mental distress than ARM (30.8%). A Chi-square test indicated that this relation between status of accompaniment (URM vs. ARM) and prevalence for mental distress was statistically significant [*χ*^2^ (1, *n *= 224) = 15.33, *p *< 0.001]. Regarding symptom severity of mental distress, RHS-15-scores of URM (Mdn = 12.04; SD = 6.32) were higher than those of ARM (*M* = 8.00; SD = 6.97). A Mann–Whitney test indicated that this difference was statistically significant (*U* =  − 2585.50, *Z* =  − 4.608 *p *< 0.001.) with a medium effect size *d* = 0.624 (95% CI 0.308 to 0.939) according to Cohen [27].

### Associated factors of mental distress in URM

The overall regression model showed that the factors *Weekly contact with a family member*, *Number of SLE, Gender, Fear of deportation, School attendance*, and *German language skills* significantly predicted the RHS-Score and accounted for 32% of its variance, *R*^2^ = 0.32, *F*_(1, 155)_ = 12.39, *p *< 0.001. Specifically, we found that *Weekly contact with a family member* was the strongest associated factor and accounted for 14% of the variance (Δ*R*^2^ = 0.14, *F*_(1, 160)_ = 26.56, *p *< 0.001), followed by *Number of SLE,* which accounted for 7% of the variance (Δ*R*^*2*^ = 0.07, *F*_(1, 159)_ = 13.89, *p *< 0.001). The items *Female gender* (Δ*R*^2^ = 0.03, *F*(_1, 158)_ = 6.23, *p *< 0.05), *Fear of deportation* (Δ*R*^2^ = 0.03, *F*_(1, 157)_ = 6.97, *p *< 0.01), and *School attendance *(Δ*R*^2^ = 0.03, *F*_(1, 156)_ = 6.77, *p *< 0.01) each accounted for 3% of the variance of the regression model. *German language skills* was found to be the weakest significant associated factor and accounted for 2% of the variance of the model (Δ*R*^2^ = 0.02, *F*_(1, 155)_ = 4.70, *p *< 0.05).

The items *Importance of religion, Duration of flight, Age, Country of origin, Time spent in host country, Having friends in the host country*, *Having a trusted person*, and *Sharing a room* did not significantly attribute to the regression model and were therefore excluded. The findings are shown in Table 2.

**Table 2:** Summary of stepwise regression analysis for variables predicting RHS-score (*n *= 172)

Variable	Model 1			Model 2			Model 3			Model 4		
	*B*	*SE B*	*ß*	*B*	*SE B*	*ß*	*B*	*SE B*	*ß*	*B*	*SE B*	*ß*
Family	− 10.52	2.04	− .37***	− 8.52	2.04	− .31***	− 7.92	2.02	− .28***	− 6.74	2.03	− 24***
SLE				2.02	.54	.27***	2.26	.54	.30***	2.08	.54	.28***
Gender							7.85	3.15	.18*	8.43	3.09	.19**
Deportation										1.68	.64	.19**
School												
Language												
*R*^2^		.14***			.21***			.24***			.27***	
Δ*R*^2^		.14***			.07***			.03*			.03**	

Variable	Model 5			Model 6		
	*B*	*SE B*	*ß*	*B*	*SE B*	*ß*
Family	− 6.49	1.99	− .22***	− 6.32	1.97	− .23**
SLE	2.02	.53	.27***	1.97	.52	.27***
Gender	9.17	3.05	.21**	8.83	3.02	.19**
Deportation	1.73	.63	.19**	1.64	.62	.18**
School	− 6.52	2.51	− .18**	− 6.79	2.48	.18**
Language	2.56	1.14	.15*	2.47	1.14	.14*
*R*^2^		.30***			.32***	
Δ*R*^2^		.03*			.02*	

Family Weekly contact with a family member, SLE Numer of stressful life events, Gender Female Gender, Deportation Fear of deportation, School School attendance, Languange German language skills, ΔR2 Change of R2

*p < .05.

**p < .01

** p < .001

## Discussion

On the basis of a large and representative study sample, we were able to add further insight in prevalences of depression and mental distress of minor refugees in Germany. Thereby, we detected significantly higher prevalence rates in URM compared to ARM. The prevalence estimates of UMR far exceed the reported prevalences in the overall minor refugees’ population [4, 6, 11]. Thus, as suggested, URM can be considered a high-risk subgroup within the minor refugee population. Therefore, a differentiation between URM and ARM in future research will most likely reduce the problem of heterogeneity in prevalences.

Furthermore, our results provide insight in influencing factors of URMs’ mental distress levels, and shed light on their vast heterogeneity of prevalence estimates. We detected six factors significantly associated with URMs mental health and sequenced them according to their explained variance. Our findings indicate that *number of SLE, female gender,* and *fear of deportation* increase the likelihood of mental distress in URM, whereas factors like *weekly contact with a family member, school attendance,* and *German language skills* decrease the risk of mental distress. Thereby, the factor *Weekly contact with a family member* accounted for the largest amount of variance and can therefore be considered as the strongest associated factor. This finding is in line with another cross-sectional study, which found fewer symptoms of depression and increased rates of cultural competences in URM, who were in frequent contact to their family members [28]. Moreover, two other cross-sectional studies reported higher rates of mental distress when family contact was lacking [29, 30]. However, a review on psychological interventions for ARM emphasizes potential burdens of frequent family contact such as feelings of overwhelming responsibility for the parents or being stressed by their parents’ traumatization [9]. Nevertheless, the authors also highlight the predominant protective effect of family contact. Our results regarding *number of SLE* and *female gender* are consistent with numerous previous findings on URM [31–35] and, thus, underline their well-established significance as associated factors for mental distress in URM [15]. The associated factor *fear of deportation*, however, lacked a comprehensive evaluation so far [15]. Similarly to our results, one large longitudinal study reported having no permanent residence status to be a risk factor for internalizing mental health problems in URM [34], whereas another longitudinal study reported no such influence [36]. The influence of school-associated factors on URM has only been investigated in one study so far, which reported a protective effect of a safe school environment on mental health of Sudanese URM [37]. However, our study is the first to show a relation between sole school attendance and URMs mental distress levels. Highly self-rated *German language skills* correlated significantly with mental distress in our study and accounted for 2% of the variance. This finding is supported by a cross-sectional study with only male URM which reported fewer symptoms of depression and PTSD for URM with high language skills. Similarly, in a longitudinal study, high levels of cultural competences had a positive effect on mental health of URM [28]. The factors *Duration of flight*, *Time spent in host country, Country of origin*, and *Age* did not show a statistically significant association to mental distress in URM. These findings are in line with the results of our systematic review, which characterized these factors as not reliable predictive due to an inconsistent study situation [15]. The factor *importance of religion* did also not attribute significantly to the accounted variance in our study. This is in contrast to overall findings of a meta-analysis on a general population, which provided evidence for a small positive effect of importance of religion on mental health [38]. However, this effect was mainly driven by studies investigating female adults, whereas our sample was primarily comprised of male adolescents. Regardless of its impact on mental health, religiousness was considered to be important by most URM (69.6%) and should therefore be acknowledged by cultural sensitive interventions and future research regarding URM.

### Limitations

For the purpose of the study, we developed a questionnaire to assess a wide range of associated factors for mental distress among minor refugees based on our previous systematic literature search. However, the response format of the *MEHIRA-SQ* was quite heterogeneous due to content-related reasons. For example, gender was assessed dichotomously, age was measured linear, and language skills were rated on a five-point-Likert scale. Furthermore, due to economic reasons, the outcome measure was solely assessed via a self-rated screening instrument. Future research might use our findings to develop a more uniformly diagnostic interview tool. Thereby, future research should investigate some factors in more depth such as accommodation type (e.g., full-care units) or perceived discrimination. Since our results are based on cross-sectional data, it is not appropriate to draw causal conclusions. Therefore, our findings should be interpreted very cautiously and need further validation by longitudinal studies. Furthermore, the comparably high prevalence rates in our study might partly be explained by our recruitment method. Since our data collection was embedded in a screening process for an RCT study, psychologically healthy minor refugees might have been less interested in participation. We tried to counteract on this effect by emphasizing the need for healthy participants prior to the screening process.

## Conclusion

Our results indicate URM to be a highly vulnerable subgroup within minor refugees which is at great risk of developing clinically relevant mental disorders. Therefore, they should receive particular attention by health services and be monitored more closely regarding their mental status. Within URM, regular family contact has shown to be strongly associated with an improved mental health. Thus, health authorities should make every effort to support family contact in URM by facilitating access to mobile contracts or if possible enhancing the family reunification process. Furthermore, supporting a rapid acquisition of language skills may improve URMs’ mental health by facilitating social integration and the use of health care services. Hence, it is of utmost importance to promote social and educational integration of minor refugees in the host countries. Since long-term pending asylum applications put further pressure on the already burdened URMs’ mental constitution, health authorities should strive for faster processing of asylum application and clarification of their residential status.

## Supplementary Information

Below is the link to the electronic supplementary material.

Supplementary file1 (PDF 440 KB)
