# Supplementary material for: Prevalences of mental distress and its associated factors in unaccompanied refugee minors in Germany
Source: Eur Child Adolesc Psychiatry. 2021 Dec 17;32(7):1211–7. doi: 10.1007/s00787-021-01926-z (PMC10276087; doi:10.1007/s00787-021-01926-z)
Supplement: Supplementary file 1 — Supplementary file1 (PDF 440 KB) [file 787_2021_1926_MOESM1_ESM.pdf]

Screening (englisch, >18J)

Patient Name:

Date of Visit:

Place of Visit:

Age:

MEHIRA – Zusatzfragebogen-Screening

SC **1. Why did you migrate / escape? *(Multiple answers possible)***

○ war
○ political or religious persecution
1 ○ natural disaster
○ social reasons (i.e. family reunification, improvement of social status etc.)
○ economic reasons (i.e. unemployement, impoverishment etc.)
○ individual reasons (i.e. better education, curiosity, adventurousness etc.)
○ other: ______________________________

2. How long you were on the run? How long did the escape last?

2 ____month ____ weeks ____days

3 **3. Have you been accompanied during your migration?**

○ yes, all the time ○ partly ○ no, not at all

4 ***if yes: who accompanied you? (Multiple answers possible)***

○ parents ○ other relatives ○ acquaintance ○ friends

4. Did you experience any of the following stresses in your life? *(mehrere Antworten möglich)*

○ lack of food or water
5 ○ physical attack (i.e. beaten, punched, attacked, stabbed, hurt or shot etc.)
○ torture
○ imprisonment
○ fear of death / agony
○ sexual violence
○ witnessing someone getting injured or killed
○ losing relatives
○ other _______________________

5. When did you migrate to Germany?

6 / / (dd/mm/yyyy)

6. Do you keep contact to your family in your country of origin?

7 ○ yes ○ no

SC

MEHIRA – Zusatzfragebogen-Screening

8

if yes: how often?

◯ every day
◯ 3-4-times a week
◯ once a week
◯ 2-3-times a month
◯ once a month
◯ less

9

7. Do you share a room?

◯yes ◯ no

10

8. Are you going to school?

◯ yes ◯ no

11

9. Is there anyone you can talk to if you don't feel well or if have any problems?

◯yes ◯no

12

10. 10. Do you have any friends in Germany?

◯yes ◯no

13

11. 11. Are you afraid of beeing deported?

◯ **yes** ◯ **no**

14

if yes, how much?

◯ a little bit
◯ moderately
◯ quite a bit
◯ very much

15

12. How would you describe your german language skills ?

◯very good ◯good ◯ medium ◯ bad ◯ very bad

16

13. How important is your religion to you?

◯very important ◯important ◯moderate important ◯ less important ◯not important

Thank you!
